# Supplementary material for: Large-scale synthetic data enable digital twins of human excitable cells
Source: eLife. 2026 Jul 23;15:RP110013. doi: 10.7554/eLife.110013 (PMC13395456; doi:10.7554/eLife.110013)
Supplement: Peer review history

# Editor report: eLife Assessment

**DOI:** 10.7554/eLife.110013.3.sa0

**Authors:** Thomas Hund

**Affiliations:** The Ohio State University United States

**Keywords:** Convincing, Important

In this **important** study, the authors present an interesting platform for digital twin construction of iPSC-CMs using an AI-based approach. The concept is timely and could have meaningful impact as the field continues to explore integration of computational and experimental models. The evidence is **convincing** overall, although additional attention to framing and calibration of claims would enhance clarity and better reflect the current level of validation.

# Referee report: Reviewer #2 (Public review)

**DOI:** 10.7554/eLife.110013.3.sa1

Summary:

The authors present a computational framework for generating "cell-specific" digital twins of human iPSC-CMs from a single optimized voltage clamp recording. Using deep learning trained on > 1 million artificial cells, the authors demonstrate that the model can infer 52 biophysical parameters governing 6 major ionic currents, and the resulting digital twins can reproduce experimentally recorded action potentials.

Comments on revised version:

The authors propose an interesting platform for digital twin construction of iPSC-CMs using an AI-based approach. However, regarding the fundamental concerns raised in the previous review round "lack of experimental validation" and "overstatement of the claims", the authors have merely added text to the "Limitations" in the Discussion, without providing any new wet-lab experimental data. This cosmetic revision fails to demonstrate the scientific validity of the platform, and the core issues remain completely unresolved.

I think the authors need to either provide substantial additional experimental data or drastically tone down the claims throughout the manuscript based on the following three major concerns.

(1) Lack of wet validation

The authors show that their AI model can infer 52 parameters from a single patch-clamp recording and reproduce the overall action potential waveform. However, the most critical validation (whether the individual ion channel parameters, such as IKr/ICaL, inferred by the AI actually match the true parameters of that specific cell) is still missing. Without a direct head-to-head comparison between the parameters inferred by the model and the exact values measured using conventional wet experiments, it is impossible to determine whether the platform is providing accurate prediction (or merely performing a curve-fitting).

(2) Absence of experimental validation for drug response simulations (Cell 1 vs. Cell 2)

In Figure 6, the authors present a simulation result where the administration of an IKr blocker (E-4031) induces EADs in the digital twin of Cell 1, but not Cell 2. However, there is absolutely no wet-lab validation for this prediction. Unless the authors actually administer the same drug to the live Cell 1 and Cell 2 from which the recordings were taken, this "computational drug response prediction" remains purely hypothetical. There is no evidence provided that the prediction accurately reflects real biological responses.

(3) Significant overstatement regarding "inter-individual variability" and "personalized medicine"

The authors state in the very first sentence of the Abstract: "Individual variability shapes how diseases manifest, how patients respond to therapy, and how rare phenotypes arise". However, this opening sentence is severely disconnected from the actual conclusions and data presented in this study. The platform can capture only "cell-to-cell variability within the same dish" (which is not even validated), and thus claiming "patient-to-patient differences" is an overstatement.

# Referee report: Reviewer #3 (Public review)

**DOI:** 10.7554/eLife.110013.3.sa2

Summary:

This work use convolution neural network to optimize a voltage clamp protocol to identify features and parameters from human pluripotent stem cell-derived cardiomyocytes.

Strengths:

The major strength is the methodology used to bridge in silico prediction of cell behavior and mechanistic insights from experimental dataset.

Comments on revised version.

As highlighted by the authors, due to the variability of the hPSC-CM model, to increase the applicability of this method, additional experimental dataset from different hPSC-CM lines would increase the translation of this approach.

I personally found that the detailed description of the methods, including the rationale of including/excluding some parameters, is extremely helpful to whoever would like to use this approach in their research.

# Author comment: Author response

**DOI:** 10.7554/eLife.110013.3.sa3

**Authors:** Pei-Chi Yang, Mao-Tsuen Jeng, Deborah K Lieu, Regan L Smithers, Gonzalo Hernandez-Hernandez, Fernando L Santana, Colleen E Clancy

**Affiliations:** University of California Davis Medical Center Davis United States; University of California Davis Medical Center Davis United States; University of California Davis Medical Center Davis United States; University of California Davis Medical Center Davis United States; University of California Davis Medical Center Davis United States; University of California Davis Medical Center Davis United States; University of California Davis Medical Center Davis United States

The following is the authors’ response to the original reviews.

**Public Reviews:**

**Reviewer #1 (Public review):**

Summary:

This study presents an interesting approach for finding electrophysiological models that match experimental patch-clamp data. The authors develop a new method for deriving optimized current clamp protocols by training a neural network on synthetic data. This optimized current clamp is then used on both computational training data and on experimental data to predict current gating and conductance parameters that correctly reconstruct the electrical phenotype.

Strengths:

(1) The fitting of gating variables through an optimized patch clamp protocol is interesting.

(2) The inclusion of experimental data is important, and the approach is shown to be effective in fitting them.

Weaknesses:

(1) Some clarity is necessary on the generation and selection of variable IPSC models. With such a large variation in so many parameters, I would expect some resulting parameters to generate non-realistic phenotypes, quiescent cells, etc. Are all 200,000 or 1,100,000 generated cells viable? Or are they selected somehow for realistic cell properties?

Thank you for this important point. We agree that broad parameter variation can generate non-physiological model behavior. Indeed, with the +/-40% perturbation range, some simulated cells produced non-realistic outputs, including quiescent behavior, and failure to generate a complete action potential. These cases were excluded from the dataset. As a result, only cells exhibiting physiologically meaningful and numerically stable behavior were retained for further analysis. We have clarified this selection procedure in the Methods section. We applied a large variation to ensure that all possible combinations and morphologies were included in the training and testing data so the model would readily ingest new data and perform robustly.

(2) The error shown in Figure 4 between different population sizes is not completely explained in the text - there seems to be a minimal difference between a population of 1,000 and 10,000, followed by a very good fit at 200,000. Is there a particular threshold that needs to be crossed where the error drops off? Related, how was the 200,000 number chosen?

Thank you for this observation. We agree that the decrease in error shows a gradual performance improvement as the population size increases, rather than a strict cutoff. As shown in Figure 4, the difference between 1,000 and 10,000 samples is small, but as we continue to increase and get to around 200,000 samples, we see strong error minimization. This indicates how much training data is needed for optimal model performance. This improvement is due to better coverage of the high-dimensional parameter space, which helps the network learn the nonlinear relationships between the parameters and outputs.

We tested a range of training data sets and found that above 200,000 training data sets, the model consistently produced low, stable errors and good test-training agreement. The test error decreased with the training error as the population size increased, indicating better generalization and suggesting that the model accurately predicts unseen data rather than overfitting to the training set.

(3) Related to the point above, the 1,100,000 population for fitting experimental data also needs a more complete explanation: how was this number chosen, and how does the error compare with the other population sizes shown in Figure 4?

Thank you for this question. We found that at a training data set size of 1,100,000 we were able to cover the large parameter space induced by +/-40% parameter perturbation. iPSC-CM measurements are known to exhibit high variability, and we wanted to capture the full range in the training data set so the model could ingest a wide range of experimental data. It is trivial to generate new training data, for example, to capture different experimental conditions like temperature differences, mutations, drugs, or ionic variability. We view this flexibility as a substantial strength of the approach. But the large perturbations we show in this study (+/-40%) allow the generation of a very broad range of cellular phenotypes while maintaining physiologically realistic ionic current properties and action potential behavior. Consistent with Figure 4, increasing population size reduces prediction error and improves generalization. The larger dataset provided more stable, accurate predictions when fitting experimental data, without evidence of overfitting.

(4) Why are the optimized current clamp protocols different between panels A and B in Figure 5? Are they somehow informed by experimental data?

Thank you for this question. The stimulation protocol used in panels A and B is identical. Panels A and B show whole-cell currents recorded under the same stimulation conditions as in Figure 3. The differences reflect variability in the underlying whole-cell ionic currents of the model cells rather than differences in the applied protocol. This is exactly the idea: the exact same protocol will generate different whole-cell currents in individual cells, but the model can find parameter sets for all of them.

(5) Figure 6D: Is the EAD risk in panel D specific to cell 1, 2, or the pooled variants of both?

Thank you for this question. We have clarified this point in the revised manuscript. The EAD risk shown in panel D is computed from the pooled variants of both Cell 1 and Cell 2, rather than being specific to either cell individually.

(6) How sensitive is the fitting to minor parameter variation? Further, if one were to pick, let's say, the next-best-fitting value, would that fall close to the best one? Is the solution found unique, or are there multiple sets with good fits?

Traditional optimization methods, such as Nelder–Mead, directly fit the model to the observed data by iteratively minimizing the error for each dataset. As a result, the solution can depend on the initial parameter guess and may converge to different local minima. In contrast, our approach trains a deep learning model on synthetic data generated from the baseline model, learning a mapping from whole-cell currents to the corresponding 52-parameter sets by minimizing prediction error. The mean squared error (MSE) decreases from approximately 10⁻² to below 10⁻³, with training and test errors overlapping closely, indicating stable training, good generalization, and accurate reproduction of the observed signals.

The model achieves very low MSE and reproduces the electrophysiological outputs with high fidelity. However, accurate reproduction of the outputs does not imply a unique parameter solution. This is illustrated in Figure S1, where baseline and predicted parameter values show close agreement overall, yet small deviations persist across parameters. This indicates that different parameter combinations can yield similar whole-cell behaviors due to parameter correlations and compensatory effects. In such cases, the model learns to predict a representative parameter set that is most consistent with the training data and loss function, rather than converging to a single unique solution within a fixed numerical tolerance.

**Reviewer #2 (Public review):**

Summary:

The authors present a computational framework for generating "cell-specific" digital twins of human iPSC-CMs from a single optimized voltage clamp recording. Using deep learning trained on > 1 million artificial cells, the authors demonstrate that the model can infer 52 biophysical parameters governing 6 major ionic currents, and the resulting digital twins can reproduce experimentally recorded action potentials.

Strengths:

The framework has clear potential for understanding cellular heterogeneity in iPSC-CMs, predicting individual drug responses, and reducing the experimental burden of multiple patch clamp protocols.

Weaknesses:

There are several concerns about the validation of the model and its clarity. First, the biological variability being modeled in this manuscript is not defined well. It is unclear whether the framework addresses cell-to-cell differences within a single differentiation batch, variability across iPSC lines, or donor-to-donor differences. This ambiguity makes it difficult to interpret what the "digital twin populations" actually represent biologically. Second, the main claim, "the digital twins enable drug testing and arrhythmia prediction that would be impractical experimentally", is not experimentally validated. For example, the E-4031 simulations predict EAD rates, but no direct experimental head-to-head comparison is provided to confirm that these predictions are accurate. Third, technical reproducibility and biological representativeness are not assessed. Single voltage clamp recordings are inherently noisy. Without knowing how much variability comes from the recording process (technical variation) vs true biological differences, it is difficult to judge whether observed "cell-specific" parameter differences are meaningful. In addition, the optimized protocol is claimed to be superior to conventional approaches, but again, no experimental comparison is shown.

The authors should address these concerns, with particular emphasis on clarifying the biological context and providing direct experimental validation. Below are detailed specific points:

(1) Ambiguous definition of iPSC-CM heterogeneity. The authors model "typical iPSC-CM heterogeneity" by varying 52 parameters +/- 40% around a baseline model (Figure 1), generating > 1 million synthetic cells. However, the manuscript does not clearly state what biological variability this model is intended to capture. Is this modeling within-line, cell-to-cell variability (e.g., cells from the same dish or differentiation batch that differ due to stochastic gene expression or maturation state)? Or is this modeling between-line or between-donor variability (e.g., genetic background differences, reprogramming efficiency)? This distinction is critical for interpretation. If the goal is to understand why different cells in the same dish behave differently, then training data should reflect that. If the goal is to compare patient lines or disease models, the framework needs validation across multiple donors or lines.

For example, the experimental validation in Figure 5 uses a single iPSC line (iPS-6-9-9T.B), but how many differentiation batches or dishes were tested, or whether cells came from the same preparation are unclear. Another example is that the wide AP diversity in the training population (Figure 1A) is impressive, but there is no demonstration that real experimental cells actually fall within this assumption range of +/- 40%.

From a biological perspective, iPSC-CMs are known to be highly heterogeneous within lines (maturation state, metabolic differences, epigenetic variation, spatial differences within the same dish, etc) and between lines (different donor/genetic background). Thus, please explicitly state whether the +/- 40% variation is intended to model within-line or between-line heterogeneity, and justify this choice with wet experiment data (or reference to experimental literature on iPSC-CM variability). Please clarify how many dishes, differentiation batches, and time points post-differentiation were used for experimental recordings (Figures 5-6). If the framework is intended to generalize across lines from different donors, please test the model on multiple independent iPSC lines (from different donors).

Thank you for this important and insightful comment. The selected ±40% range was chosen to broadly explore all physiologically plausible electrophysiological behaviors, not to match a specific experimental distribution. Our goal was to cover enough behaviors for the model to learn a reliable mapping between responses and ionic parameters.

We recognize that this approach does not explicitly account for variability between lines or donors. We have a current project focused on extending the framework to include multiple iPSC-CMs from patient donors, but given that the model framework successfully reproduces such a broad range of cell phenotypes, we feel confident that it will readily apply to different genetic backgrounds from patient-specific cells. This study is underway.

We have updated the manuscript to clarify how the modeled variability is interpreted and added a discussion of these limitations. Furthermore, we clarified the experimental conditions, such as the number of differentiation batches and recording settings, in the revised Methods section.

(2) Biological representativeness of single-cell measurements.

The framework generates digital twins from single voltage clamp recordings. The patch clamp recordings in iPSC-CMs are subject to substantial technical variability. The manuscript does not address a fundamental question: "How representative are the measurements from a single cell on the dish (or line)?" In other words, if I measure one cell from a dish of a million cells, does that cell's digital twin tell me something about the dish as a whole, or just about that one cell? The manuscript presents Cell 1 and Cell 2 (Figures 5-6) as distinct individuals, but it's unclear whether these differences reflect true biological heterogeneity or simply sampling variability. I think the authors should perform replicate recordings on multiple cells (e.g., > 10 cells) from the same dish (same differentiation batch) and quantify how much the inferred parameters vary, and then compare between lines.

Thank you for this important comment. We agree that the representativeness of single-cell measurements and the impact of technical variability are important considerations in interpreting the results. In this study, the framework is designed to generate digital twins that reflect the electrophysiological properties of individual recorded cells, rather than to directly represent the behavior of the entire cell population within a dish.

As such, differences observed between Cell 1 and Cell 2 are intended to reflect variability at the single-cell level, which may arise from a combination of biological heterogeneity and experimental variability. We agree that systematic replicate recordings across multiple cells are valuable to quantify the relative contributions of biological and technical variability, and to assess the consistency of inferred parameters. However, this is beyond the scope of the current study. We have added clarification in the manuscript to explicitly state this limitation and to outline this as an important direction for future work.

(3) No experimental validation of the main claim that in silico populations can replace wet experiments.

The most exciting claim in the manuscript is that digital twins enable drug testing and arrhythmia prediction "at scale" without requiring hundreds of patch clamp experiments. Specifically, the authors show that in silico populations derived from two experimental cells (Figure 6C) predict dose-dependent EAD incidence for the IKr blocker E-4031 (Figure 6D), with ~3% of cells showing EADs at 50 nM.

However, this prediction is not validated experimentally. If I actually patch 20-30 real iPSC-CMs and apply 50 nM E-4031, will ~3% of them show EADs, as the model predicts? Without this validation, I think the drug testing framework is purely hypothetical. The model may be internally consistent (e.g., Cell 1's twin behaves differently from Cell 2's twin), but there is no evidence that these in silico populations reflect real biological variability in drug response. Please provide experimental validation that justifies the prediction by digital twins.

Thank you for this important comment. We agree that experimental validation of population-level drug response will be valuable for establishing the quantitative accuracy of the predicted EAD incidence. The E-4031 simulations are intended as a proof-of-concept illustrating how the framework can identify susceptible subpopulations and quantify relative proarrhythmic risk *in silico*. We agree that direct comparison with large-scale experimental datasets is a key next step, and we are working hard to get the study funded so that we can perform those experiments and bring this technology to scale.

(4) Experimental validation and head-to-head comparison of optimized protocol.

The authors claim that their deep learning-optimized voltage clamp protocol (Figure 3, Figure 4A) is superior to conventional approaches, but they have not validated this experimentally by doing a head-to-head comparison. The manuscript does not compare the optimized protocol to any published voltage clamp designs. If the optimized protocol is genuinely easier to implement and more informative than existing approaches, this would be a major practical advance. But without side-by-side comparison, it is impossible to judge whether the optimization made a real difference.

Thank you for your comment. We agree that comparing directly with traditional voltage-clamp protocols through experiments would be useful. In this study, our main aim was to show that the optimized protocol enhances parameter inference within the modeling framework, not to prove experimental superiority. We have clarified this point in the revised version.

**Reviewer #3 (Public review):**

Summary:

This work uses a convolutional neural network to optimize a voltage clamp protocol to identify features and parameters from human pluripotent stem cell-derived cardiomyocytes.

Yang et al. introduce an innovative experimental framework that integrates computational modeling and deep learning to generate a digital twin of human pluripotent stem cell-derived cardiomyocytes (hPSC-CMs).

Strengths:

The major strength is the methodology used to bridge in silico prediction of cell behavior and mechanistic insights from the experimental dataset.

The approach used in this study represents a significant step toward precision medicine by enabling in silico prediction of cellular behavior and mechanistic insight from experimental datasets. The study addresses an important and timely challenge in stem cell-based and personalized medicine, and the authors compellingly leverage state-of-the-art methods alongside strong expertise in computational modeling and cardiac electrophysiology

Weaknesses:

While the overall approach is highly compelling and the potential impact is substantial, there are two areas where clarification and refinement, particularly in the phrasing and framing used throughout the manuscript, would further strengthen the work.

(1) While the overall goal of the study is compelling, the manuscript would benefit from clearer articulation of how the proposed framework is intended to be used in practice. In particular, it is not entirely clear whether the authors envision this approach as:

(a) a method to extract population-level trends that, when paired with biological data, enhance statistical power and interpretability, or

(b) a strategy capable of constructing a population-based model from limited single-cell recordings. If the latter is intended, additional guidance on the number of action potentials required per cell and the assumptions underlying this extrapolation would greatly clarify the scope and applicability of the method.

Thank you for this thoughtful comment. We agree that the intended use of the framework should be more clearly articulated. In this study, we generate a large synthetic population of iPSC-CM models by varying 52 biophysical parameters governing key ionic currents. A neural network is trained on simulated whole-cell current responses to learn a mapping between current profiles and model parameters. Experimental recordings are then used as inputs to this trained model to infer ionic parameters, rather than directly fitting the model to data. This enables individual recordings to be interpreted within a large, physiologically plausible parameter space and supports population-level analysis of electrophysiological variability. The primary goal of the framework is therefore to facilitate mechanistic interpretation of variability and relate experimental observations to underlying ionic currents. But the longer-term intended goal is to develop digital twins from patient-derived cell lines and then use populations constructed from patient-specific digital twins to screen therapeutics and identify arrhythmia marker vulnerability in a very thorough and high-throughput way. We have clarified this in the revised manuscript.

(2) The manuscript would also benefit from a clearer explanation of how electrophysiological heterogeneity observed in hPSC-CMs is linked to inter-patient variability. Although the authors state that this framework can be generalized to compare patient-specific hiPSC-CM lines, it remains unclear how this generalization is achieved, given the substantial sources of variability intrinsic to hiPSC-CMs (e.g., batch effects, reprogramming strategy, differentiation protocol, and maturation state). As acknowledged by the authors, addressing this level of variability likely requires large datasets; further clarification of how the proposed approach mitigates or accommodates these challenges would strengthen the translational claims.

Below are my suggestions that could help strengthen the claims in the manuscript:

(1) Adding a dedicated section describing the electrophysiological phenotype of the hPSC-CMs used in this study would help justify the choice of the underlying ionic model and the selection of the six ion currents analyzed. These currents are not only developmentally regulated but may also vary substantially across different hPSC-CM lines, which has implications for generalizability.

Thank you for this important suggestion. We agree that providing additional context on the electrophysiological phenotype of the hPSC-CMs strengthens the rationale for both the underlying ionic model and the selection of currents analyzed.

We have expanded the Methods section to clarify this point. Briefly, the ionic currents were selected based on the Kernik-Clancy iPSC-CM model developed in our prior work, which was specifically designed to capture the range of electrophysiological variability observed within an iPSC-CM cell line using a population-based framework. In this model, variation in key ionic conductances is sufficient to reproduce the diversity of action potential morphologies, spontaneous activity, and repolarization dynamics commonly reported experimentally, while avoiding non-physiological behaviors.

Accordingly, we focused on six primary ionic currents that are known to play dominant roles in shaping action potential characteristics and variability in iPSC-CMs. This selection reflects a balance between model parsimony and physiological relevance, enabling the framework to capture the expected spectrum of variability within a given cell line. We also note that the framework is extensible, and additional currents or alternative parameterizations can be incorporated to account for differences across cell lines, donors, or experimental conditions in future studies. See updated discussion.

(2) If feasible, inclusion of patch-clamp data from an additional hPSC-CM line would significantly strengthen the claim that this framework can harmonize and generalize across datasets and cell sources.

Thank you for this helpful suggestion. We agree that adding data from more hPSC-CM lines would improve the framework's generalizability. In this work, our goal was to show that the digital twin framework is data-driven and can easily be expanded to include more hPSC-CM lines, allowing for cross-line comparisons in future studies. We have clarified this and included a discussion of this limitation in the revised manuscript. We are currently seeking funding for patient-specific lines as well to allow scalability.

(3) The authors note that the experimental cells exhibited high variability in action potential morphology. This is an important observation that directly supports the motivation for the study and should be explicitly presented, even if only in the supplementary materials.

Thank you for this suggestion. We agree that explicitly showing the variability in experimental action potential morphology strengthens the motivation for this study. We have now added a section in the discussion discussing this and referencing the many prior studies that focused on iPSC-CM variability, including the studies upon which our initial model (Kernik-Clancy) was based.

(4) In the hERG-blocker experiments, further clarification is needed regarding the biological relevance of the reported 3% incidence of early after depolarizations (EADs). Additionally, an interrupted sentence in this section makes it unclear whether the goal is to demonstrate that the digital twin can capture rare arrhythmic risk events or whether the digital twin is necessary to determine whether this level of risk is clinically meaningful.

Thank you for this important comment. We agree that more clarification is needed on the ~3% EAD incidence and the digital-twin role. This analysis aims to show that electrophysiological variability can create a small, susceptible subpopulation under drug effects, not to set a clinical risk threshold. The observed ~3% EAD incidence reflects the emergence of such a susceptible subpopulation under hERG block. While relatively small, this fraction is important because it arises from modest, physiologically plausible variation in ionic properties and would be difficult to capture using single-cell or small-sample approaches. As described in the Discussion, this variability-driven emergence of EADs provides a quantitative measure of proarrhythmic risk at the population level. The digital-twin framework enables systematic identification and quantification of these rare events, linking cell-level variability to population-level responses. We have revised the manuscript to clarify this point.

(5) The manuscript states that some action potentials were excluded from the experimental dataset. A brief explanation of the exclusion criteria, along with guidance on how to distinguish high-quality from low-quality recordings, would improve transparency and reproducibility.

Thank you for this comment. We agree that the definition of failed recordings should be clarified. We have now specified the exclusion criteria in the Methods section.

**Recommendations for the authors:**

**Reviewer #1 (Recommendations for the authors):**

(1) It would be helpful if the network cartoon in Figures 2 and 3 were replaced with a simplified sketch of the actual neural network used.

Thank you. We now have new figures 2 and 3.

(2) Subsection title for the Introduction has a typo.

Thank you. We have fixed it.

**Reviewer #2 (Recommendations for the authors):**

(1) Technical quality control criteria are not specified.

The Methods section states that "any incomplete or failed recordings were excluded," but does not define what constitutes a failed recording. The criteria could be subjective.

Thank you for pointing this out. We agree that the definition of failed recordings should be clarified. We have now specified the exclusion criteria in the Methods section.

“Recordings were excluded if they exhibited no spontaneous firing, abnormally slow firing rates, or failed to capture a complete action potential waveform. These criteria were applied consistently across all recordings.”

(2) "Cell-specific" may overstate the claim.

The term "cell-specific digital twins" (title, throughout) implies that the inferred parameters reflect the true biological state of each cell. However, parameters are derived only from curve-fitting to electrophysiological data and do not reflect other biological components (e.g., gene expression, contractility, calcium handling, metabolism, etc). Please consider rephrasing to "electrophysiology-based digital twins", "voltage clamp-matched digital twins", etc.

Thank you for this important comment. We agree that the term “cell-specific” could be interpreted as implying a complete representation of the biological state of each cell. We have also adjusted the wording in relevant sections to avoid over-interpretation.

**Reviewer #3 (Recommendations for the authors):**

(1) I would add the list of the 52 parameters in the method section/SI and not just in the reference. Additional justification of why the perturbation was set as +/- 40% for the 52 parameter or +/- 20% for the EAD population would also help.

Thank you for this helpful comment. We have included model equations and highlighted the 52 parameters in the Supplementary Information and provided additional justification in the Methods.

(2) In Figure 1B, might be helpful to add the axis of the Vm instead of the dotted line indicating 0 mV to show differences in the diastolic potential.

Thank you! We have now updated Figure 1B.

(3) Figure 1C-I might be more impactful to show traces from the AP shown in Figure B to reinforce the impact of a single current in the AP shape.

We have now updated Figure 1C-I to include traces from the AP shown in Figure 1B.
